# Post-Bariatric Plastic Surgery: Abdominoplasty, the State of the Art in Body Contouring

**DOI:** 10.3390/jcm11154315

**Published:** 2022-07-25

**Authors:** Payam Sadeghi, Daniela Duarte-Bateman, Wanyan Ma, Ryan Khalaf, R’ay Fodor, Gorizio Pieretti, Feliciano Ciccarelli, Hamed Harandi, Roberto Cuomo

**Affiliations:** 1 Plastic Surgery Department, Cleveland Clinic Main Campus, 9500 Euclid Ave, Cleveland, OH 44195, USA; duarte.daniela0723@gmail.com; 2 Geisinger Commonwealth School of Medicine, Scranton, PA 18510, USA; wma@som.geisinger.edu; 3 School of Medicine, Case Western Reserve University, Cleveland, OH 44106, USA; rxk646@case.edu; 4 Cleveland Clinic Lerner College of Medicine, Cleveland, OH 44195, USA; fodorr@ccf.org; 5 Surgical and Dental Sciences, Multidisciplinary Department of Medical, University of Campania Luigi Vanvitelli, 81100 Caserta, Italy; dott.goriziopieretti@gmail.com (G.P.); felicianociccarelli@libero.it (F.C.); 6 “Villa Dei Fiori” Clinic, 80011 Acerra, Italy; 7 Department of Surgery, St. Elizabeth’s Medical Center, Tufts University School of Medicine, Boston, MA 02111, USA; hamed.harandi.md@gmail.com; 8 Plastic and Reconstructive Surgery Unit, Department of Medicine, Surgery and Neuroscience, “Santa Maria alle Scotte” Hospital, University of Siena, 53100 Siena, Italy; robertocuomo@outlook.com

**Keywords:** abdominoplasty, post-bariatric plastic surgery, body contouring, weight loss, American Society of Plastic Surgeons

## Abstract

Due to the increased prevalence of obesity in the last decades, bariatric surgery has been on the rise in recent years. Bariatric surgery is a compelling option for weight loss in obese patients with severe obesity-related comorbidities or for whom lifestyle modifications have proven ineffective. Redundant skin following significant weight loss is a common occurrence affecting up to 96% of patients who undergo bariatric surgery, negatively impacting physical and psychosocial health and detracting from activities of daily living. Statistics of the American Society of Plastic Surgeons show that 46,577 body contouring procedures were performed after massive weight loss in the USA in a 2020 report. Abdominoplasty, a well-established cosmetic surgery procedure for improving body contour, is performed by removing excess skin and fat from the abdominal wall and thereby restoring musculofascial integrity and skin elasticity, resulting in a more ideal body shape and increasing quality of life. Although abdominoplasty is a safe procedure, it has been associated with a higher complication rate compared with other body-contouring procedures. Technologic advances over the past decade have been developed as non-invasive alternatives or adjunctive tools to surgery to enhance cosmetic results and minimize complications. New energy-based technologies may supplant invasive surgery for mild to moderate skin laxity and/or diminish the extent of surgery and resulting scars. Plastic surgeons play a significant role in improving the quality of life of patients who suffer from obesity and underwent bariatric surgery. We are deeply convinced, however, that the advancement of knowledge and research in this field will determine the introduction of new technologies and custom-made techniques. This advancement will reduce the complication rate with a rapid reintegration of the patient into the world of work and resumption of daily activities.

## 1. Introduction

According to the WHO, the prevalence of overweight adults in the world is 39% (39% of men and 40% of women) while the prevalence of obese adults (BMI > 30) is 13% (11% of men and 15% of women) [1]. In 2001, the WHO introduced the term “globesity” by combining the words globe and obesity with the purpose of defining and highlighting the ever-growing obesity epidemic worldwide [2]. Obesity is fast becoming one of the biggest health care complications in the world and is related to substantial complications and mortality [3]. Globally, the frequency of obesity and overweight in the adult population is estimated at 604 million, and long-standing obesity has been associated with metabolic, cardiovascular, physical, and psychological problems [3,4]. According to prediction models, by the year 2030, the rate of obesity in adults may be around 42% [5].

This trend has led to the development and improvement of disciplines such as food science and bariatric surgery. Bariatric surgery is a compelling option for weight loss in obese patients with severe obesity-related comorbidities or for whom lifestyle modifications have proven ineffective [6]. The usage of bariatric surgery in the severe obesity treatment has a number of advantages, including maintainable weight loss, improvement or elimination of some metabolic diseases, and improving life expectancy [7]. A recent systematic review and meta-analysis on ten studies reported that bariatric surgery reduces the incidence of Major Adverse Cardiovascular Events (MACE) in patients with obesity and cardiovascular diseases [8]. The combination of these advantages with the sustained decrease in complications has led to significant growth in appeal for bariatric surgery worldwide [9]. It is estimated that the frequency of bariatric surgeries is about 256,000 each year [10]. Weight loss following bariatric surgery is both significant and sustainable, with the majority of weight loss occurring during the first two years [6,11]. Although patients generally begin regaining weight during the second postoperative year, long-term weight gain is minimal, with weight stabilizing over the course of several years [6].

There are now several options for bariatric surgery, including sleeve gastrectomy (SG), Roux-en-Y gastric bypass (RYGB), vertical-banded gastroplasty (VBG), adjustable gastric banding (ABG), and biliopancreatic diversion with duodenal switch (BPD/DS) [12]. Of these, SG is the most common, accounting for approximately 61% of the 252,000 bariatric procedures performed annually in the United States [6]. Although the magnitude of weight loss varies by procedure, bariatric surgery is proven to be successful for long-term weight loss, with all procedures outperforming the most effective nonsurgical weight loss interventions [6]. For example, post-RYGB patients lose approximately 30% of their baseline weight one year post-surgery, after which weight remains stable, even after 10 years [13]. Outcomes of SG are similar to those of RYGB [6]. In general, SG and RYGB are the most successful procedures in terms of total weight loss relative to baseline.

Although the majority of patients who undergo bariatric surgery are satisfied with their treatment outcomes, these procedures are not without long-term side effects [14]. It was suggested that patients who underwent bariatric surgery are at risk of nutrient deficiencies, including vitamins B12, B1, folate, A, D, C, and K, as well as trace minerals, such as iron, zinc, selenium, and copper, and they may need life-long extra doses of prophylactic supplementation to preserve optimal status of micronutrients. It is suggested that all bariatric surgery patients should be monitored regularly for nutrient levels of the serum beginning three months after surgery and periodically afterwards [15]. Gastroesophageal reflux, treatment failure requiring operative revision, incisional hernia, nutritional deficits, stomach cancer, gallbladder disorders, liver necrosis, pancreatic disorders, acute kidney failure, and redundant skin are reported as complications of bariatric surgery [16]. Redundant skin following significant weight loss is a common occurrence affecting up to 96% of patients who undergo bariatric surgery, negatively impacting physical and psychosocial health and detracting from activities of daily living [17,18,19]. The effects of redundant skin are so pervasive and consequential that the majority of patients also eventually desire a body contouring (BC) operation following bariatric surgery [19]. Abdominoplasty, brachioplasty, thighplasty, mastopexy, lower body lift, abdominal panniculectomy, torsoplasty, and cruroplasty are all commonly sought BC procedures which are effective for removing redundant skin [19]. Of these procedures, abdominoplasty is most commonly sought [20]. The aim of this manuscript is to describe the state of the art of abdominoplasty through a literature analysis.

## 2. Ethical Approval

**Figure 1:**
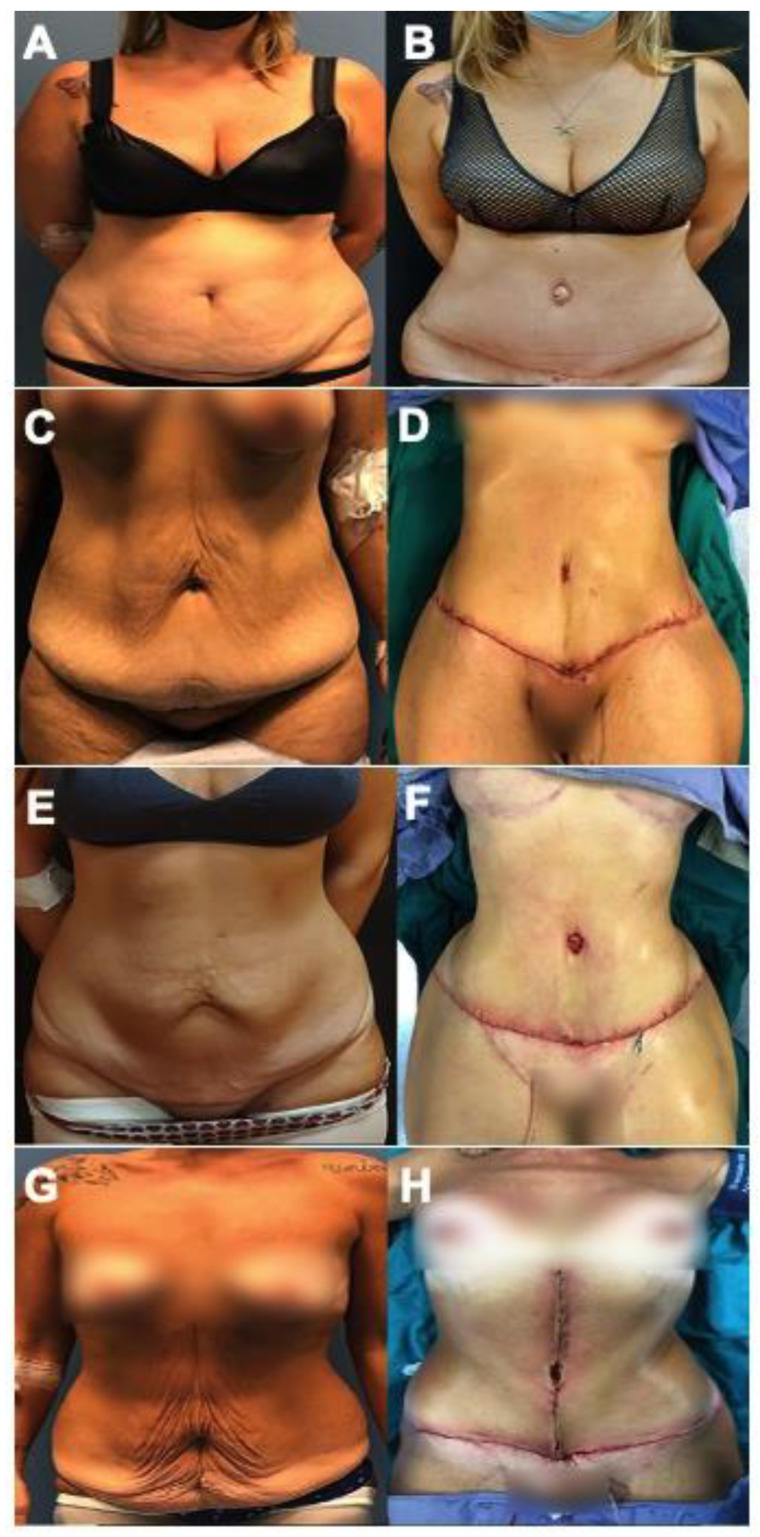
Anteroposterior (AP) view: before (**A**,**C**,**E**,**G**) and after (**B**,**D**,**F**,**H**) the patients underwent abdominoplasty. (**A**,**B**): Female, 43 years old, underwent traditional abdominoplasty after sleeve gastrectomy and 18 kg of weight loss. (**C**,**D**): Female, 36 years old, abdominoplasty after gastric mini-bypass and 36 kg of weight loss. (**E**,**F**): Female, 32 years old, abdominoplasty after sleeve-gastrectomy and 28 kg of weight loss. (**G**,**H**): Female, 52 years old, abdominoplasty with vertical scar after sleeve-gastrectomy and 43 kg of weight loss.

**Figure 2:**
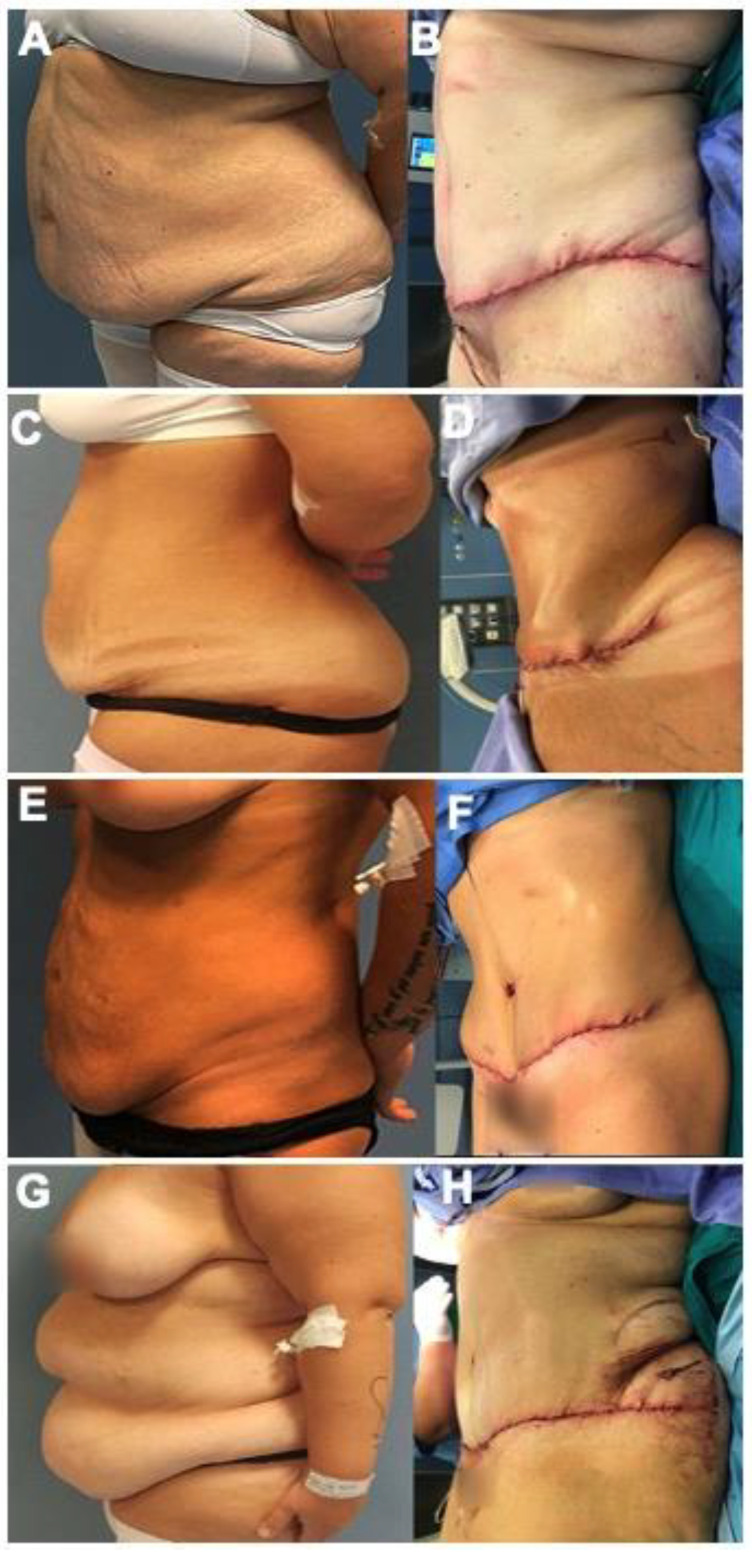
Lateral view: before (**A**,**C**,**E**,**G**) and after (**B**,**D**,**F**,**H**) photos of the patients underwent high superior tension abdominoplasty. (**A**,**B**): Female, 52 years old, submitted to traditional abdominoplasty after gastric balloon and 19 kg of weight loss. (**C**,**D**): Female, 39 years old, abdominoplasty and abdominal wall reparation for umbilical hernia after gastric mini-bypass and 26 kg of weight loss. (**E**,**F**): Female, 40 years old, abdominoplasty after sleeve-gastrectomy and 32 kg of weight loss. (**G**,**H**): Female, 50 years old, lipo-abdominoplasty after sleeve-gastrectomy and 49 kg of weight loss.

**Figure 3:**
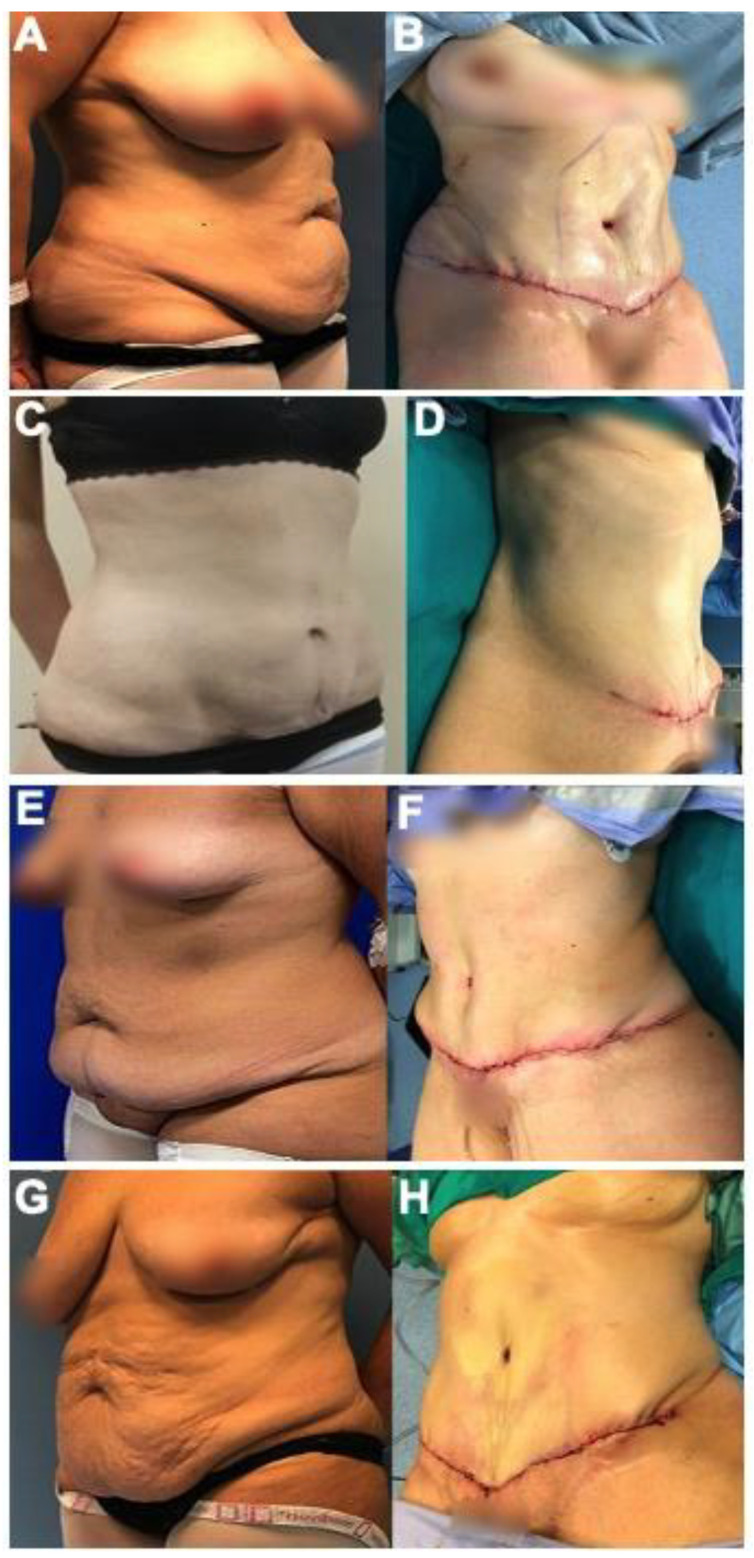
AP-lateral view: (**A**,**C**,**E**,**G**) and after (**B**,**D**,**F**,**H**) photos of the patients underwent lipo-abdominoplasty and high superior tension using radiofrequencies energy for dissection. (**A**,**B**): Female, 44 years old, submitted to traditional abdominoplasty after gastric mini-bypass 41 kg of weight loss. (**C**,**D**): Female, 29 years old, abdominoplasty after diet and 18 kg of weight loss. (**E**,**F**): Female, 40 years old, abdominoplasty after sleeve-gastrectomy and 55 kg of weight loss. (**G**,**H**): Female, 59 years old, abdominoplasty after mini-bypass and 45 kg of weight loss.

**Figure 4:**
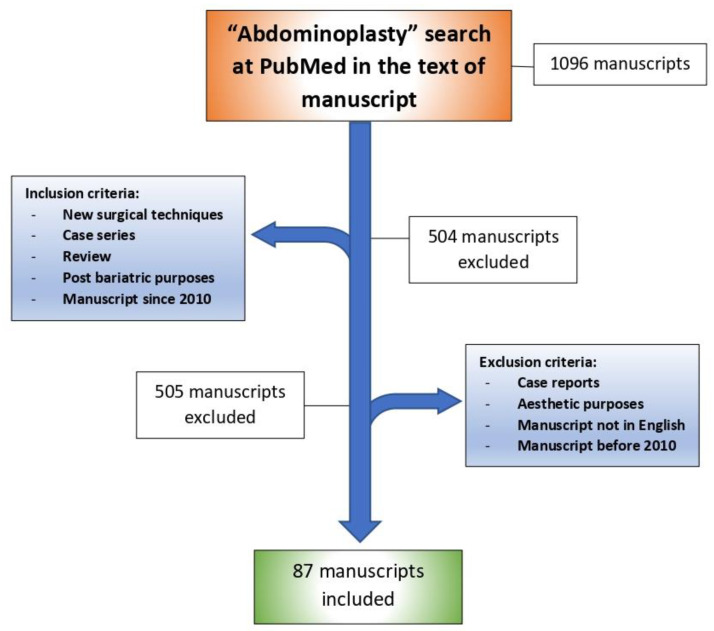
Searching process flow chart displaying inclusion and exclusion criteria.

The following is a retrospective/narrative review manuscript. No clinical trial registration was required to write this manuscript. The approval of Ethical Committee was obtained. We included the before and after photos from 12 female patients (Figure 1, Figure 2 and Figure 3) who underwent post-bariatric abdominoplasty in Plastic and Reconstructive Surgery Division, Villa Dei Fiori Clinic, Acerra, Italy. The age range was 29–59 years old (mean: 43 years old) and the patients were informed completely that their photos were taken for the purpose of publication and the written specific consents were signed, accordingly. The searching process is displayed in a flow chart including the inclusion and exclusion criteria (Figure 4).

## 3. Abdominoplasty in Post-Bariatric Patients

Due to the increased prevalence of obesity in the last decades, bariatric surgery has been on the rise in recent years [21]. Clinical treatment is effective for most obese patients, but in the case of the severely obese, bariatric surgery is the most effective method [22]. However, patients are then often left with the functional and aesthetic sequelae of the massive weight loss from the bariatric surgery. The excess skin can cause limitations in mobility and exercise, difficulty in performing personal hygiene, skin irritation and infections, psychological and emotional distress, and social discomfort. Therefore, functional reconstructive surgery after bariatric surgery is routinely offered. Statistics of the American Society of Plastic Surgeons show that 46,577 body contouring procedures were performed after massive weight loss in the USA in 2020 report [23]. One of these procedures is abdominoplasty, a well-established cosmetic surgery procedure for improving body contour. This is done by removing excess skin and fat from the abdominal wall and thereby restoring musculofascial integrity and skin elasticity, resulting in a more ideal body shape and increasing quality of life [21,24]. Although abdominoplasty is a safe procedure, it has been associated with a higher complication rate compared with other body-contouring procedures [13,25,26,27].

It is known that obese patients undergoing abdominoplasty have a significantly increased risk of developing complications compared to non-obese patients [13,28,29,30,31]. Efforts have been made to identify other risk factors in the post-bariatric population that increase the rate of complications from the procedure, but further studies are needed. De Paep et al. conducted the largest retrospective analysis of exclusively post-bariatric abdominoplasty cases, with a total of 898 patients. An overall complication rate of 29.8% was found. Type I complications (minor wound problems) occurred in 15.8% (*n* = 140). Type II complications requiring medical intervention occurred in 10% (*n* = 90). Type III complications occurred in 36 patients, with re-intervention for wound problems (*n* = 16), seroma (*n* = 16), umbilical necrosis (*n* = 4), and bleeding (*n* = 6). Five patients developed deep venous thrombosis or pulmonary embolism. The weight of tissue resected, the interval between bariatric and body contouring surgery, preoperative BMI, male gender, diabetes mellitus type 2, and smoking were important predictors for developing complications [21].

Schlosshauer et al. also conducted a study of a large sample of the post-bariatric population (*n* = 406) and reported a complication rate of 42%. They concluded that post-bariatric patients with a BMI ≥ 30 kg/m^2^ at the time of surgery are at an increased risk for wound-healing problems [32]. Furthermore, Schlosshauer et al. also found an increased risk of complications associated with increasing age (risk of complications increases 1.270 times every 10 years), gender (male patients have 2.111 times the risk of complications compared with female), and resection weight (every 500 g increases the risk of complications 1.099 times) [33]. Donato et al. also concluded male gender was an independent risk factor for total complications after controlling for other variables as well as major and minor complications due to this population’s increased age, smoking prevalence, number of comorbidities, higher BMI, and American Society of Anesthesiologists (ASA) classification [28]. Lievain et al. compared the complications of abdominoplasty of post-bariatric patients (*n* = 114) versus non-bariatric patients (*n* = 124) and observed that the complication rate in post-bariatric patients was significantly higher (55.3% against 26.6%) with problems mainly related to healing [34].

A comparison of complication rates between different studies is challenging due to the lack of standardization in the reporting methods [21]. However, knowledge from published large-cohort studies should be used by surgeons to identify higher-risk patients and educate them on their risk for undergoing post-bariatric abdominoplasty.

## 4. Different Techniques in Abdominoplasty

**Figure 5:**
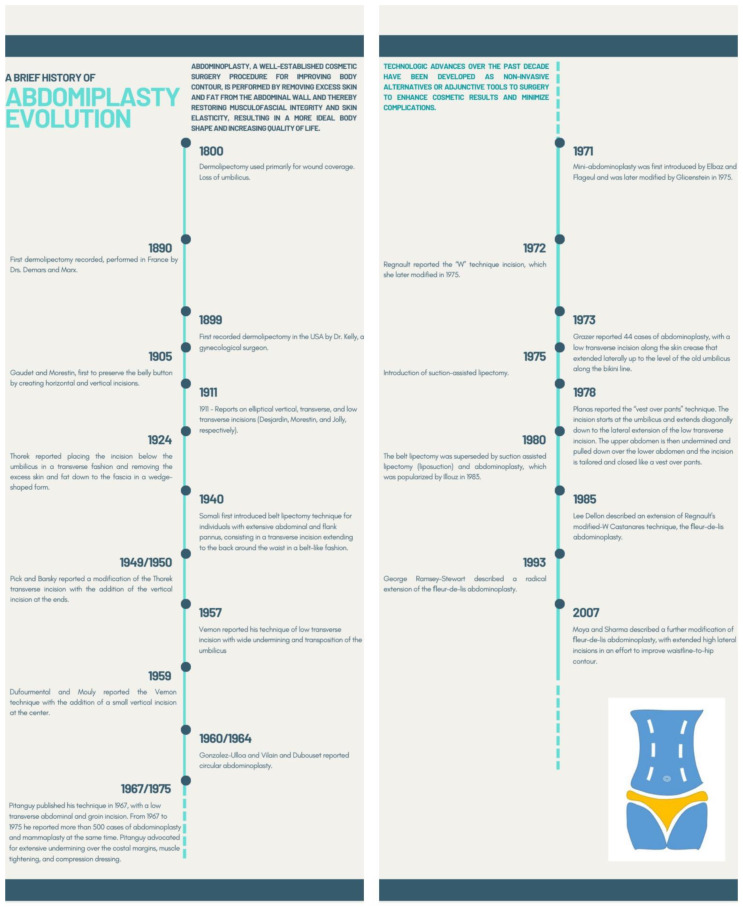
Timeline illustration showing the evolutionary history of abdominoplasty techniques.

The techniques of abdominoplasty were first described in general surgery in an attempt to facilitate the approach to umbilical hernias. They were performed mainly in obese patients and primarily were limited to the skin and excess fat resection, hernias, and diastases [35]. With further diffusion of the technique, many plastic surgeons have modified the technique with the objective of increasing the safety of the procedure and achieving the most natural results. The evolutionary history of abdominoplasty [36] is illustrated in Figure 5.

In post-bariatric patients, contouring procedures should not commence until weight loss is complete and has been stable for a minimum of six months. This will usually not occur until at least 12 months after bariatric surgery [37]. The abdomen is often the area of most concern for patients as the excess skin hangs over the pubis and thighs, creating major hygiene and mobility problems [37]. As post-bariatric patients lose weight overall, the remaining skin tends to be loose with poor quality and tone. Patients often have several areas of concern (such as the lower back, breasts, chest, arms, thighs, face, and neck), so traditional abdominoplasty in these patients is rarely recommended. Other procedures, such as the circumferential body lift (also known as the belt lipectomy), are more commonly recommended as they target these other areas that traditional abdominoplasty fails to address.

In 1961, Gonzalez-Ulloa described the circumferential abdominoplasty, and later Hunstad popularized the procedure for obese patients [38,39]. The belt lipectomy, in addition to allowing the surgeon to address the laxity of the abdominal wall musculature and fascia, allows the excess skin from the lateral thighs, hips, buttocks, and lower back to be released and excised. This contributes to a better contour of the torso following major weight loss [40]. In patients who have skin laxity limited to the anterior trunk and abdomen without a back or buttock component, traditional abdominoplasty can be considered. This procedure addresses the anterior abdominal contour and pannus with the possibility of repositioning and insetting the umbilicus. When preformed in post-bariatric patients, the procedure is typically converted to an “extended abdominoplasty” by expanding the incisions laterally and posteriorly to avoid a dog-ear deformity [37].

An alternative technique used to address the lax and expanded horizontal component of the abdominal wall is the fleur-de-lis technique. The name comes from the way the pattern is marked (in the shape of a fleur-de-lis), and it has both a horizontal and vertical component. The tissue within the marks is resected, and the incisions are closed in an inverted T formation, at the expense of an additional scar. This allows the surgeon to gather excess horizontal tissue at the flanks and draw it medially, thereby creating an aesthetically pleasing waistline. The complication rate of the fleur-de-lis has been shown to be comparable to that of the traditional abdominoplasty [41].

In addition to abdominoplasty, liposuction can be used to achieve better results. It has been proven that adding liposuction to an abdominoplasty does not put patients at additional risk [42]. However, when suctioning areas of the body which will not have a concomitant skin resection, skin tone and quality must be carefully evaluated as the procedure can result in additional excess ptotic skin when skin fails to retract over time [37]. Shestak described a modification of the belt lipectomy in which the skin excision is carried to the posterior hip area with the remainder of the posterior truncal contouring performed with liposuction [43]. Abdominoplasty, with or without liposuction, is a safe procedure [42]. In post-bariatric patients with substantial skin excess, more than one abdominoplasty procedure might be necessary to achieve optimal results [44]. Adequate surgical planning and precautions should be taken to ensure patient safety and optimal results.

## 5. Complications in Abdominoplasty

BC is considered as a safe procedure. However, it was shown that the rate of complications postbariatric body contouring surgical was 31.5% and the seroma was the most common complication. The BMI ≥ 30 kg before body contouring and the higher weight of resected tissue were revealed to be associated with a higher risk of complications [45]. BC complication rates vary by procedure (Table 1 and Table 2), but the overall complication rate is around 13%, with wound dehiscence and seroma among the most common complications [20].

Abdominoplasty has been the most popular BC choice post bariatric surgery, but it is not without its risks and complications, some of which may be exacerbated in this complex and unique patient population. These can range from minor problems with wound healing to major, potentially life-threatening complications requiring medical intervention. Certain risk factors in patient characteristics associated with higher rates of complications have been identified, such as BMI, smoking, gender, age, resection weight, etc. [32]. Although more research and data are needed to corroborate, standardize, and establish causality with these risk factors, surgeons can still use this extant knowledge to identify patients at higher risk and council them to achieve the most optimal outcome. Abdominoplasty among aesthetic procedures is one with a relatively high complication rate. Using the CosmetAssure database, Winocour reported an overall higher rate of complications in abdominoplasty relative to other aesthetic procedures (4.0% versus 1.4%) [24]. Retrospective studies have reported abdominoplasty complication rates from 9.7% to 37.4% [46,47,48].

Although obesity has been reported as a risk factor for complications, the effects of major weight loss for the post-bariatric patient is somewhat controversial. In a study of 90 patients who underwent abdominoplasty, Vastine et al. reported 80% of obese patients had complications compared to 33% in nonobese patients but found no effect of gastric bypass surgery on increased complications [31]. However, Greco et al. reported a significant increase in wound complication after abdominal contouring in weight loss surgery patients (41% versus 22%) compared to patients who did not undergo weight loss surgery by univariate methods of analysis [49]. Although Greco et al. subsequently discuss how multivariate analysis revealed prior weight loss surgery was not an independent risk factor, they do note its importance in preoperative considerations. Similarly, Staalesen and colleagues reported higher early abdominoplasty complication rates in post-bariatric patients compared to patients who did not undergo bariatric surgery (48% versus 29%) but were unable to find predictive factors [50]. As reported separately by Chetta and Pajula, there were no difference in complication rate after abdominal contouring procedures in patients with different methods of weight loss (diet/exercise versus bariatric surgery), suggesting that bariatric surgery itself did not increase complication risk [51,52]. Several other studies report varying complication rates among post-bariatric patients [46,53,54,55].

In a recent meta-analysis, Marouf and Mortada report the most common type of complication in post-bariatric patients after abdomen contouring as seroma (weighted rate 13.3–16.4%), followed by wound dehiscence (9.6%), infection (4.3%), hematoma (1.4–3.2%), fat necrosis (0.5%), skin necrosis (0.3%), deep vein thrombosis (0.3%), and pulmonary embolism (0.3%) [45]. Despite the low rate of thromboembolic events, they may have serious sequelae and prove fatal.

Post-bariatric abdominoplasty patients are a unique subset of patients who require careful pre-operative considerations to manage safety and minimize complications. There may exist varying risk factors and a fluctuating prevalence of comorbidities, such as nutritional deficiencies, incisional scarring, and residual diseases related to obesity. In a review article, Love and colleagues discuss how bariatric surgery may lead to iron deficiency through altered iron absorption and decreased red meat consumption due to intolerance [56]. Similarly, Slater et al. report fat malabsorption and deficiencies in fat soluble vitamins (vitamin A, vitamin D, and vitamin K) after biliopancreatic diversion [57]. As such, it is important to consult a nutritionist prior to abdominoplasty in the post-bariatric patient. Residual scarring from previous bariatric surgeries may also impair wound healing through sacrificed blood supply and devascularized regions. Shermak reported a relationship between any abdominal scar and any complication, as well as increased frequency in wound healing with abdominal scars [58,59]. Furthermore, pre-operative planning should consider the findings that a larger amount of abdominal tissue removed during surgery has been associated with increased tissue necrosis and wound healing complications [45,60]. Longer procedure times have also been associated with higher incidence of complications [61]. Given the massive weight loss patients undergo the year following bariatric surgery, van der Beek et al. underscore the importance of maintaining stable weight close to normal prior to body contouring surgery to minimize risk of complications [62].

In a retrospective review of 144 abdominoplasty patients, Sirota reported no association between type of bariatric surgery (sleeve gastrectomy, laparoscopic adjustable gastric band, and gastric bypass) and incidence of complications [55]. Post-bariatric patients with major weight loss may undergo a fleur-de-lis abdominoplasty to adequately address their contour deformities, and studies have shown similar rates of complications compared to traditional abdominoplasty techniques [41]. Oftentimes, the post-bariatric patient presents with multiple regions of concern due to major weight loss, and the plastic surgeon may consider abdominoplasty combined with other body contouring procedures. Although total complication rates were higher in combined procedures, Coon et al. reported a similar number of complications per procedure that would be expected had the operations been performed separately [63]. Coon et al. also reported addition of other procedures did not increase abdominoplasty complication rate, which has been corroborated by other studies [46,63].

In summary, the post-bariatric patient population may require special pre-operative considerations to minimize abdominoplasty complications. Considerations include nutritional status evaluation, comorbidity considerations, prophylaxis of thromboembolic events, and multiple procedures or staged approaches (Table 1 and Table 2).

**Table 1:** Different abdominoplasty techniques’ benefits and complications.

Authors.	Benefits	Complications	Study Design	Number of Participants
**Gilmartin et al.** [22]	Excess skin limits mobility and exercise, causes difficulty performing personal hygiene, skin irritation and infections, psychological and emotional distress, and social discomfort. Body contouring surgery such as abdominoplasty can improve self-esteem and mood.	Long-lasting scarring	Retrospective	20
**De Paep et al.** [21]	Restores musculofascial integrity and skin elasticity.	29.8% complication rate: minor wound problems, complications require medical intervention, seroma, umbilical necrosis, bleeding, DVT/PE	Retrospective	898
**Schlosshauer et al.** [33]	N/A	42% complication rate. Increased risk of wound healing problems. Increased complications associated with age, male gender, resection weight.	Retrospective	406
**Donato et al.** [28]	N/A	Male gender is risk factor for complications	Retrospective	4369
**Lievain et al.** [34]	N/A	Complication rate significantly higher in post-bariatric patients, mainly healing problems	Retrospective	238
**Vastine et al.** [31]	N/A	80% obese patients had complications vs 33% in nonobese. No effect of gastric bypass on complications.	Retrospective	90
**Greco et al.** [49]	N/A	Significant increase in wound complications for post-bariatric patients	Retrospective	89
**Staalesen el al** [50]	N/A	Significant increase in wound complications for post-bariatric patients	Retrospective	161
**Chetta et al.** [51]	N/A	No difference in complication rate	Retrospective	307
**Pajula et al.** [52]	N/A	No difference in complication rate	Retrospective	158
**Neaman and Hansen** [46]	N/A	Variable complication rates	Retrospective	206
**de Kerviler et al.** [53]	N/A	Variable complication rates	Retrospective	104
**Brito et al.** [54]	N/A	Variable complication rates	Retrospective	191
**Sirota et al.** [55]	N/A	Variable complication rates	Retrospective	144
**Marouf and Mortada** [45]	N/A	Most common complications in post-bariatric patients: seroma, wound dehiscence, infection, necrosis, DVT, PE	Systematic review and meta-analysis	522

**Table 2:** Current techniques and complications.

Authors	Current Techniques	Complications
**Gonzalez-Ulloa** [38]	Circumferential abdominoplasty	N/A
**Hunstad** [39]	Circumferential abdominoplasty	N/A
**Friedman and Michaels** [41]	Fleur-de-lis	Complication rate comparable to traditional abdominoplasty.
**Shestak** [43]	Belt lipectomy modification	N/A

## 6. Recent Technologies in Post-Bariatric Body Contouring

Technologic advances over the past decade have been developed as non-invasive alternatives or adjunctive tools to surgery to enhance cosmetic results and minimize complications (Table 3). New energy-based technologies may supplant invasive surgery for mild to moderate skin laxity and/or diminish the extent of surgery and resulting scars [64].

To improve post-bariatric patients’ skin tone and quality, various energy-based technologies (e.g., ultrasound, radiofrequency, electromagnetic) have been introduced into clinical practice as described by Zocchi and Duncan [65,66]. In particular, the concept of mixing lipo-assisted-liposuction and radiofrequency was introduced to obtain better tissue tightening, achieving greater skin surface area reduction. This was an important key point: the use of an energy source to improve skin retraction. The superficial application of ultrasound was improved by Jewell et al. in 2002 using a pulsed low-power ultrasound small probe known as VASER (vibration amplification of sound energy at resonance) [67]. The idea of modifying the fat layer introduced the new concept of the goal of enhancing the detail of the muscular plane. This was difficult to obtain because often the superficial fat layer obscures the muscular detail. In order to improve the aesthetical outcomes, Hoyos and Millard introduced high definition liposculpture [68].

Technological advancements introduced radiofrequency (RF) for the contraction of collagen and neocollagenesis, resulting in skin and fascia contraction. Cook et al. reviewed over 745 patients treated with bipolar radiofrequency as an adjunct to face and body contouring and found overall high patient satisfaction and minimal adverse events [69]. Zendejas et al. compared the use of a percutaneous radiofrequency device in adjunct to standard liposuction to suction-assisted lipectomy (SAL) alone, demonstrating significant postoperative correction of skin laxity by the use of radiofrequency than with SAL alone [70]. Pierazzi et al. studied a new radiofrequency device called “Ligasure Impact” to reduce complications after the detachment of the fat layer from the muscular fascia, demonstrating a reduction in seroma and bleeding risk in the treated group [71].

More recently, a novel high-intensity focused electro-magnetic (HIFEM) technology has been used to improve muscular development and tone. In a prospective, multi-center, non-randomized pilot study, Kinney and Lozanova demonstrated via MRI simultaneous muscle growth, fat reduction, and reduced abdominal separation at two months and six months post treatments using a HIFEM device [72]. Ultrasound-assisted lipoplasty (UAL) is another technique that removes fat through a fat emulsification process termed “cavitation” and it is extensively used to debulk fat, undermine flaps, and harvest adipose tissue [73].

**Table 3:** Recent technology utilization in abdominoplasty.

Authors	Assistive Technologies	Complications
**Vidal et al.** [42]	Liposuction	Does not put patients at additional risk.
**Herman et al.** [37]	Liposuction	Could introduce excess ptotic skin.
**Zocchi** [65]	Ultrasonic-assisted lipoplasty	N/A
**Irvine Duncan** [66]	Radiofrequency-assisted liposuction	N/A
**Jewell et al.** [67]	VASER	No major complications
**Hoyos and Millard** [68]	VASER-assisted high definition liposculpture	6.5% incidence of seromas, 2.94% incidence of port skin burns
**Cook et al.** [69]	Bipolar RF	Minimal adverse events
**Pierazzi et al.** [71]	Ligasure Impact	Reduction in seroma and bleeding risk
**Kinney and Lozanova** [72]	High-Intensity Focused Electro-Magnetic (HIFEM)	Muscle growth, fat reduction, reduced abdominal separation
**Rohrich et al.** [73]	Ultrasound-assisted lipoplasty	N/A
**Schlosshauer et al.** [74]	Pulsed electron avalanche knife (PEAK)	Less tissue damage, lower complication rate, fewer seroma

Different technical devices for dissection in abdominoplasty have been introduced and comparatively assessed to minimize perioperative and postoperative complications in post-bariatric patients. Schlosshauer et al. evaluated the effects of a low-thermal plasma dissection device (PEAK, pulsed electron avalanche knife, PlasmaBlade) in comparison with conventional electrosurgery in a randomized clinical study [74,75]. In the PEAK PlasmaBlade group, there was significantly less tissue damage, a lower total complication rate, and fewer postoperative seroma resulting in faster wound healing.

The improvement of surgical techniques for weight loss have led to the definition of a “post-bariatric” population, one which includes patients with large amount of skin requiring body reshaping. Complications related to excess skin may include intertriginous rashes, interference with ambulation, ulcerations, and problems in daily activities, and may adversely affect the quality of life [76]. The complications related to redundant skin cannot be fully resolved through physical exercise, diet, or modifications in lifestyle [77]. Therefore, post-bariatric body contouring surgery appears to be an ideal and feasible solution for removing the extra skins and adipose tissues [78].

As a direct consequence, post-bariatric plastic surgery is receiving substantial input in standardization of procedures. Many techniques have been described to obtain favorable results, and researchers are also investigating medical procedures to increase positive outcomes. Clarity rooted in scientific evidence is further needed on this topic. The role of the plastic surgeon is crucial in redefining and restoring (in terms of form and function) the ideal anthropomorphic proportions and geometries of the patient. This is in essence a restoration of individual integrity and a redefinition of body harmony.

## 7. Impact of Abdominoplasty on Health-Related Quality of Life (HRQoL)

Abdominoplasty was demonstrated to be related to decreased secondary weight regain after bariatric surgery [79]. It may have been associated with bodily satisfaction and improved physical activity, or biological response to adipose tissue reduction. The adipose tissue is shown to be a metabolic active organ and secretes several hormones and cytokines that are related to the regulation of appetite, inflammation, and energy metabolism [80,81]. Therefore, removing excess skin and adipose tissue can have a helpful biological effect on weight loss and decrease secondary weight retain after bariatric surgery. Improvements in health-related quality of life (HRQoL) was reported in most post-bariatric patients who underwent body contouring surgery [76]. An improvement in physical activity was demonstrated after BCS in patients after massive weight loss [82]. It was reported that abdominoplasty can decrease the depression symptoms in post-bariatric patients [83]. While aesthetic improvement is one primary outcome of BC, there are many other important benefits, including reduced BMI, reduced incidence of skin rash and infection, and increased quality of life [17,18]. A significant improvement in the general understanding of personal appearance and the social and psychological aspects of patients’ life after body contouring post-bariatric surgery has been reported [84].

Despite a majority of patients post bariatric surgery seeking BC, only 5–7% actually undergo BC [19]. This discrepancy may be driven by a combination of factors, such as operative risk and the relative financial inaccessibility of BC as many insurance companies still view it as a purely cosmetic procedure [85]. Only 7% of bariatric surgeons guide their patients to consult with a plastic surgeon, and most patients who underwent bariatric surgery are not aware of the plastic surgery services [86].

The majority of patients report long-term satisfaction with BC following bariatric surgery, with the highest rates of satisfaction for procedures involving the breast, hips, and buttocks, and the lowest rates of satisfaction for procedures affecting the thighs [87]. Improvement in weight loss was observed in terms of BMI change, total body weight loss, and excess weight loss in patients who underwent BC surgery after bariatric surgery [19]. Approximately 85% of patients report improved self-esteem following bariatric surgery and BC compared to 48% for patients who underwent bariatric surgery without BC [19]. Improvements in career advancement, as well as relationship status post-BCS, were also reported [88]. Improving the HRQoL as a result of BCS increases patients’ capability to advance weight loss via increasing motivation levels as well as the desire to attain or preserve a better appearance [19]. BC following bariatric surgery also improved outcomes of obesity-related comorbidities. Overall, BC is a compelling surgical option following bariatric surgery, but further research regarding uptake and outcome metrics are needed to optimize results for patients with redundant skin.

## 8. Conclusions

Post-bariatric plastic surgery is a constantly evolving discipline. This has allowed both functional and aesthetic results to constantly improve, along with increasing patient demand. Despite new technologies and the advancement in knowledge of lymphatics, the post-bariatric patient remains a complex patient due to skin excess and malabsorptive bariatric surgery, which contribute to altering the trophic state of the skin. This in turn produces interventions with a high risk of complications, such as seromas and bleeding. Plastic surgeons play a significant role in improving the quality of life of patients who suffer from obesity and elect to undergo bariatric surgery. We are deeply convinced, however, that the advancement of knowledge and research in this field will determine the introduction of new technologies and custom-made techniques. This advancement will reduce the complication rate with a rapid reintegration of the patient into the world of work and resumption of daily activities. 

## Data Availability

Not applicable.
